# New drugs, new toxicities: severe side effects of modern targeted and immunotherapy of cancer and their management

**DOI:** 10.1186/s13054-017-1678-1

**Published:** 2017-04-14

**Authors:** Frank Kroschinsky, Friedrich Stölzel, Simone von Bonin, Gernot Beutel, Matthias Kochanek, Michael Kiehl, Peter Schellongowski, on behalf of the Intensive Care in Hematological and Oncological Patients (iCHOP) Collaborative Group

**Affiliations:** 1 Dresden University Hospital, Medical Department I, Fetscherstr. 74, 01307 Dresden, Germany; 2 Department for Hematology/Oncology/Stem Cell Transplantation, Hannover Medical School, Hannover, Germany; 3 Department I of Internal Medicine, University Hospital Köln, Köln, Germany; 4 Medical Department I and Stem Cell Transplant Center, Hospital Frankfurt/Oder, Frankfurt/Oder, Germany; 5 General Hospital Department of Medicine I, Medical University of Vienna, Vienna, Austria

**Keywords:** Cancer, Targeted therapy, Immunotherapy, Toxicity, Interdisciplinary management

## Abstract

Pharmacological and cellular treatment of cancer is changing dramatically with benefits for patient outcome and comfort, but also with new toxicity profiles. The majority of adverse events can be classified as mild or moderate, but severe and life-threatening complications requiring ICU admission also occur. This review will focus on pathophysiology, symptoms, and management of these events based on the available literature.

While standard antineoplastic therapy is associated with immunosuppression and infections, some of the recent approaches induce overwhelming inflammation and autoimmunity. Cytokine-release syndrome (CRS) describes a complex of symptoms including fever, hypotension, and skin reactions as well as lab abnormalities. CRS may occur after the infusion of monoclonal or bispecific antibodies (MABs, BABs) targeting immune effectors and tumor cells and is a major concern in recipients of chimeric antigen receptor (CAR) modified T lymphocytes as well. BAB and CAR T-cell treatment may also be compromised by central nervous system (CNS) toxicities such as encephalopathy, cerebellar alteration, disturbed consciousness, or seizures. While CRS is known to be induced by exceedingly high levels of inflammatory cytokines, the pathophysiology of CNS events is still unclear. Treatment with antibodies against inhibiting immune checkpoints can lead to immune-related adverse events (IRAEs); colitis, diarrhea, and endocrine disorders are often the cause for ICU admissions.

Respiratory distress is the main reason for ICU treatment in cancer patients and is attributable to infectious agents in most cases. In addition, some of the new drugs are reported to cause non-infectious lung complications. While drug-induced interstitial pneumonitis was observed in a substantial number of patients treated with phosphoinositol-3-kinase inhibitors, IRAEs may also affect the lungs.

Inhibitors of angiogenetic pathways have increased the antineoplastic portfolio. However, vessel formation is also essential for regeneration and tissue repair. Therefore, severe vascular side effects, including thromboembolic events, gastrointestinal bleeding or perforation, hypertension, and congestive heart failure, compromise antitumor efficacy.

The limited knowledge of the pathophysiology and management of life-threatening complications relating to new cancer drugs presents a need to provide ICU staff, oncologists, and organ specialists with evidence-based algorithms.

## Background

Treatment of cancer using pharmacological approaches has changed extensively during the past two decades. Antineoplastic chemotherapy classically targets various steps of cell proliferation, such as DNA formation and function or the mitosis spindle. The treatment is commonly given intravenously in a cyclic schedule and causes side effects such as nausea, emesis, hair loss, as well as “bad blood counts”.

Long years of basic and clinical research have resulted in a switch from this repetitious iatrogenic intoxication of patients and their tumors to more targeted antineoplastic “snipers”, which eliminate tumor cell populations effectively and with milder side effects. Monoclonal antibodies (MABs) such as rituximab or cetuximab target more specific tumor cell antigens, the former targeting the CD20 protein in malignant lymphoma, the latter targeting epidermal growth factor receptor (EGFR) in colon cancer. The suppression of the blood supply, and therefore the nutrition of growing tumor nodes, was accomplished with bevacizumab, an antibody which neutralizes angiogenetic cytokines (vascular endothelial growth factor (VEGF)). These bespoke MABs initiated a new era of cancer drugs

Only a few years later, the adoption of tyrosine kinase inhibitors (TKIs) began with the use of imatinib in the treatment of chronic myeloid leukemia (CML). This established the principle of killing tumor cells by interrupting intracellular signals, which are essential for cell proliferation and survival. While imatinib was designed to interact with a highly disease-specific BCR-ABL oncoprotein, other inhibitors target more physiological pathways which are less specific but upregulated in malignant cell populations. Numerous inhibitors with different efficacies have been developed and approved for various hematological and solid tumors in recent years. The exclusively oral and continuous administration of TKIs represent an improvement in patient comfort and an important difference between TKIs and their counterparts, the classic cytotoxic drugs and MABs.

The rising stars in cancer treatment are approaches by which the patient’s immunological self-defense is strengthened in a feasible and effective manner. Bispecific antibodies (BABs), chimeric antigen receptor (CAR) T cells, and checkpoint inhibitors attack cancer cells by activating immune effectors and/or decreasing their tolerance. However, while earlier cancer drugs cause adverse events by compromising defense mechanisms, the new classes of immune therapeutics may induce overwhelming inflammatory responses and autoimmunity.

All of the new approaches substantially have improved the outcome of cancer patients in clinical trials and daily practice. Despite this, we must also consider and learn to navigate some qualitatively new toxicities. This review will summarize potentially life-threatening complications caused by new cancer agents and the strategies to manage or to prevent them.

## New agents, new toxicities

Compared to classic chemotherapy, the new cancer drugs and technologies are generally less toxic and more comfortable to the patient. Unspecific side effects relating to constitutional diseases, gastrointestinal (GI) symptoms, mucositis, and myelosuppression are commonly mild or lacking. The specific events depend on the biological target; life-threatening complications often result from infections, inhibition of angiogenetic pathways, severe inflammatory syndromes, and autoimmune disorders. An overview of the main toxicities caused by different agents and approaches is presented in Table 1. The severity of side effects after administration of anticancer drugs is characterized with the Common Terminology Criteria for Adverse Events (CTCAE) scale, which is regularly updated by the National Cancer Institute. Unspecific and organ-related adverse events are graded into different categories according to their severity: 1, mild; 2, moderate; 3, severe; 4, life-threatening or disabling; 5, fatal. The most recent 4.0 version of the CTCAE scale is available in detail at http://ctep.cancer.gov/protocolDevelopment/electronic_applications/ctc.htm. This review will focus on severe and life-threatening (CTCAE grade ≥3) events.

**Table 1:** Different classes of new cancer drugs, frequently used agents, and main toxicities

Agent	Target	Indications	Toxicities
Monoclonal antibodies			
Rituximab Ofatumumab Obinutuzumab	CD20	B-cell lymphomas and leukemias	CRS Immunodeficiency
Trastuzumab	HER2neu	Breast cancer	Cardiac disease
Cetuximab	EGFR	Colorectal cancer	Diarrhea Exanthema
Bevacizumab	VEGF	Colorectal cancer Breast cancer Renal cell cancer NSCLC	Hypertension GI bleeding or perforation Thromboembolism
Ramucirumab	VEGFR	Gastric cancer	
Tyrosine kinase inhibitors			
Imatinib Dasatinib	BCR-ABL	CML ALL	Pleural/pericardial effusions Pulmonary hypertension
Ponatinib			Thromboembolism
Erlotinib	EGFR	NSCLC Pancreatic cancer	Exanthema, diarrhea GI bleeding or perforation
Idelalisib	PI3K	B-cell lymphoma	Pneumonitis Colitis, hepatosis
Trametinib	MEK	Melanoma	Diarrhea, edema Decrease of LVEF
Aflibercept Axitinib	VEGF VEGFR	Colorectal cancer Renal cell cancer	Hypertension GI bleeding or perforation Thromboembolism PRES
Sorafenib Sunitinib Pazopanib	Multiple kinases	Renal cell cancer GIST Soft tissue sarcoma	Decrease of LVEF Hypertension
Bispecific antibodies (BAB)			
Blinatumomab	CD3/CD19	ALL B-cell lymphomas	CRS Neurotoxicity (e.g., convulsions) Liver toxicity (transaminitis)
Checkpoint inhibitors			
Ipilimumab	CTLA-4	Melanoma	IRAEs: Diarrhea, colitis Hypophysitis Immunhepatitis Polyarthritis
Nivolumab Pembrolizumab	PD-1	Melanoma NSCLC RCC Hodgkin’s lymphoma	
Cellular treatments			
CAR T cells	CD19	ALL B-cell lymphomas	CRS Neurotoxicity (e.g., convulsions, encephalopathy, or ischemia)

*CRS* cytokine-release syndrome, *VEGF* vascular endothelial growth factor receptor, *VEGFR* vascular endothelial growth factor receptor, *HER* human epidermal growth factor receptor, *GI* gastrointestinal, *NSCLC* non-small cell lung cancer, *RCC* renal cell cancer, *LVEF* left ventricular ejection fraction, *CML* chronic myeloid leukemia, *ALL* acute lymphoblastic leukemia, *EGFR* epidermal growth factor receptor, *PI3K* phosphoinositol-3 kinase, *MEK* MAP (mitogen-activated protein) kinase/ERK (extracellular signal-regulated kinase) kinase, *PRES* posterior reversible encephalopathy syndrome, *GIST* gastrointestinal stromal tumors, *CTLA-4* cytotoxic T-lymphocyte-associated protein 4, *PD-1* programmed death receptor 1, *IRAEs* immune-related adverse events, *CAR* chimeric antigen receptor

## Cytokine-release syndrome

Cytokine-release syndrome (CRS) is a potentially life-threatening systemic inflammatory reaction which is observed after infusion of agents targeting different immune effectors. Affected patients mostly develop fever, chills, hypotension, and tachycardia during or immediately after drug administration. Furthermore, the syndrome may cause a broad spectrum of constitutional and organ-related disorders, as well as blood test abnormalities (Table 2). Because events appear during or after first exposure to a “new” drug, a differentiation to anaphylaxis may be difficult. There are few allergy-specific symptoms such as urticaria or glottis edema which may guide an allergic diagnosis here.

**Table 2:** Main symptoms of cytokine-release syndrome

Constitutional	
Fever, chills, headache, asthenia, myalgia, arthralgia, back or abdominal pain	
Organ related	
Oliguria, bronchospasm, dyspnea, hypotension, tachycardia, arrhythmia, confusion, erythema, urticarial reaction, pruritus	
Lab tests	
Hypokalemia, increased urea, decreased glomerular filtration rate, altered blood counts and/or coagulation tests, elevation of C-reactive protein and/or procalcitonin	

CRS is driven by an increase of inflammatory cytokines which are released after the activation and cytotoxic damage of monocytes, macrophages, and different lymphocyte populations; current models show extensively high levels of interleukin (IL)-6 to have a central role in pathophysiology [1].

In this context, CRS has been known to be a consequence of OKT3 administration, which was formerly used prophylactically as an anti-CD3 antibody to prevent graft-versus-host disease in hematopoietic stem cell transplantation.

The syndrome became more relevant to patients and hematologists when the use of rituximab was established in the treatment of CD20-positive B-cell malignancies. The majority of events after the administration of MABs can be managed by antipyretics, antihistamines, corticosteroids, adequate fluid load, as well as cardiopulmonary monitoring and oxygen supplementation; only rarely do patients need to be admitted to the ICU for vasopressor support or hemofiltration. The incidence of serious drug-related adverse events was reported to be 1.4% among 36,000 patients treated with rituximab between 1997 and 1999 [2]. Grade 3 or 4 infusion-related reactions have also been reported in trials with the more recently developed anti-CD20 antibodies ofatumumab and obinutuzumab [3, 4] and in rare cases of solid cancer patients after administration of cetuximab or trastuzumab [5, 6].

Blinatumomab belongs to a new class of agents working as an engager of T-cell activity via binding to CD19 and CD3 (BAB). The drug is approved for relapsed or refractory B-precursor acute lymphoblastic leukemia. The treatment regimen requires a 4-week continuous infusion. Whereas CRS was found as the dose-limiting toxicity in early clinical trials, the incidence of this complication could be reduced by modification of the administration schedule and incremental dosage increase [7]. Severe (grade 3) CRS occurred in 2% of 189 patients who received blinatumomab for approved indication in a large phase 2 study [8].

Severe or fatal CRS reactions during cellular immunotherapy with CAR T cells led to further investigation of these complications and the development of more detailed management algorithms. Grade ≥3 fever, hypotension, or hypoxia was reported in up to 80, 40, and 15% of treated patients, respectively [9]. Further organ toxicities may affect the kidneys, liver, central nervous system (CNS), GI, and musculoskeletal system. Monoclonal antibodies against IL-6 receptors such as tocilizumab represent a therapeutic option for the intensivist to neutralize the key mediator and to interrupt the inflammatory process. Due to its high costs, as well as potential severe adverse events, including infections, reactivation of viruses, or tuberculosis and hepatotoxicity, treatment using tocilizumab should be limited strictly to critically ill patients. The National Cancer Institute (NCI) recommendation for the administration of tocilizumab in patients with CAR T-cell-associated CRS presented in Table 3 may also assist in treatment decisions in other types of IL-6-associated syndromes.

**Table 3:** NCI recommendation for the use of tocilizumab in patients with CAR T-cell-associated CRS (according to [9])

Tocilizumab 4 to 8 mg/kg i.v. (1-h infusion, maximum 800 mg)	
(1) Decrease of LVEF <40% assessed by echocardiogram	
(2) Increase of creatinine >2.5-fold compared to baseline	
(3) Norepinephrine support (>2 μg/min) for 48 h since start of vasopressors (even if non-continuous administration)	
(4) Decrease of systolic blood pressure <90 mmHG despite vasopressor support	
(5) Severe dyspnea potentially requiring mechanical ventilation	
(6) APTT >2× UNL	
(7) Persisting elevation (>5× UNL) of creatinine kinase longer than 48 h	

*CRS* cytokine release syndrome, *CAR* chimeric antigen receptor, *i.v.* intravenously, *LVEF* left ventricular ejection fraction, *UNL* upper normal limit, *APTT* activated partial thromboplastin time

## Central nervous system events

Beside CRS, treatment with BABs and CAR T cells may also be compromised by CNS toxicity. These events may manifest at any time during CRS or as a singular complication [9, 10]. Therefore, staff must be made aware that an intervention with BABs or CAR T cells may potentially necessitate intubation and mechanical ventilation for airway protection, as well as antiepileptic therapy in patients with seizures [9].

A considerable proportion of patients (>50%) who received blinatumomab in a phase 2 trial for acute B-lymphoblastic leukemia experienced neurologic events such as tremor, encephalopathy, cerebellar alteration, or seizures [8]. Thirteen percent of the events were classified as severe or life-threatening. Grade ≥3 neurotoxicity was reported in 13 of 27 patients in an early clinical trial with acute lymphoblastic leukemia patients undergoing CAR T-cell therapy [11]. Recently, cases of lethal cerebral edema in those patients were observed as well [12]. The pathophysiology of these neurotoxic effects is still unclear but, as in CRS, inflammatory cytokines seem to be involved [13].

Other catalysts for neurologic disorders in these patients must also be excluded, which requires extensive diagnostic work-up. This might include applying CNS-imaging procedures, cerebral computed tomography, contrast-enhanced cerebral magnetic resonance imaging, cerebrospinal fluid analyses, and electroencephalography along with thorough neurological examination.

Differential diagnosis may include infectious causes such as encephalitis due to herpes virus species, focal infections such as toxoplasmosis, primary CNS hemorrhage due to altered coagulation cascades, or, vice versa, clotting in CNS vasculature due to thrombophilia and secondary hemorrhage. Furthermore, CNS toxicities might be accelerated by applied co-medications (as a *last-straw* effect) with known potential to lower the seizure threshold, such as quinolones, or those which cause seizures, such as the classic cytotoxic drug busulfan. Immunosuppressive agents such as cyclosporine that might be applied sequentially or simultaneously can cause posterior reversible encephalopathy syndrome [13].

The conclusion of these analyses is crucial, because while the application of steroids will relieve CNS symptoms without interrupting therapy and may even prevent new onset after discontinuation, it will also impair the immune effects mediated by T cells and therefore interfere with the therapeutic approach itself. Accompanying treatment with either benzodiazepines or phenytoin is common practice in patients who receive busulfan. In contrast, there is currently no consensus on CNS prophylaxis, clinical monitoring, or emergency backup in recipients of CAR T cells or BABs. In recent phase 2 and phase 3 trials applying a CD19-directed BAB, no CNS prophylaxis was given. However, in case of any neurologic event, dexamethasone was administered at up to 24 mg/day, tapering incrementally over the following 4 days. When patients developed grade ≥3 neurotoxicity, the BAB was stopped immediately. Additional diagnostic measures in this scenario included cerebrospinal fluid assessment with cytology, cell count, and virus-PCR analyses (for HSV1/2, HHV6, JC virus, and adenovirus) [8, 14].

For CAR T cells the administration of tocilizumab in case of neurotoxicity is not recommended. An improvement of neurotoxicity was not observed in affected patients after the administration of this anti-IL6 antibody [1, 9]. The lacking efficacy might be attributable to its poor ability to penetrate the blood–brain barrier. Similar to BABs, dexamethasone is recommended in cases of CAR T-cell-induced neurotoxicity at or above grade 3 (with the exception of headaches lasting more than 24 h), grade 4 neurotoxicities (regardless of duration), and for any seizures. The current recommended dosage of dexamethasone is 10 mg i.v. every 6 h until neurotoxicity has improved to at least grade 1, or until at least eight doses have been administered [9].

## Immune-related adverse events

**Fig. 1:**
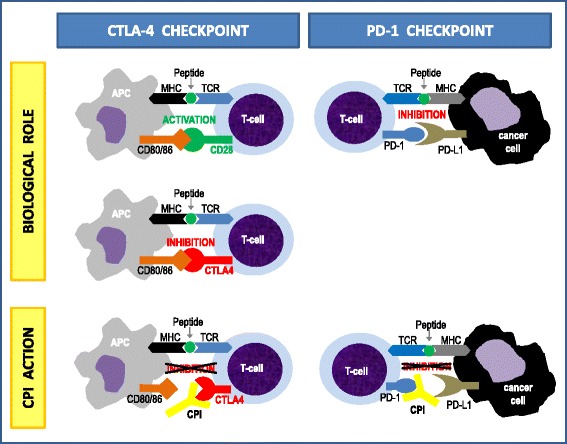
Immune checkpoints: physiological function and mode of action of inhibiting monoclonal antibodies (checkpoint inhibitors). *CTLA-4* cytotoxic T-lymphocyte-associated protein 4, *PD-1* programmed death receptor 1, *PD-L1* ligand of PD-1, *CPI* checkpoint inhibitor(s), *APC* antigen-presenting cell, *MHC* major histocompatibility complex, *TCR* T-cell receptor

For decades, intensive research was focused on improving the immune system’s innate ability to fight against cancer cells. Ipilimumab [15] was the first player in a new class of so-called “checkpoint inhibitors”, which stimulate cellular immune effectors by blocking inhibitory signals. The physiological role of immune checkpoints such as CTLA-4 (cytotoxic T-lymphocyte-associated protein 4), PD-1 or PD-2 (programmed death receptor-1 and -2), and their ligands is to limit immune reactions in order to avoid tissue damage and to allow tolerance. In various tumors these mechanisms are also used by cancer cells to overcome host defense barriers. Clinical trials with these new drugs in Hodgkin’s lymphoma and solid cancers like melanoma, non-small cell lung cancer, renal cell cancer, and colorectal cancer have shown impressive effects on patient outcome. Figure 1 illustrates biological functions of immune checkpoints and therapeutic interactions with their new inhibitors.

However, although the “inhibition of inhibition” stimulates the immune system, it can attenuate tolerance and may cause overwhelming inflammation, tissue damage, and autoimmunity. These immune-related adverse events (IRAEs) were reported in up to 85% of patients after treatment with ipilimumab [16] and up to 70% after treatment with inhibitors of the PD1 axis [17]. The main target tissues are in the GI tract, liver, skin, and endocrine system [18]. The frequency of severe, life-threatening, or even fatal (grade 3, 4, or 5) events is higher after ipilimumab (10–40%) compared to nivolumab or pembrolizumab (<5%) [17, 19]. Combining ipilimumab and nivolumab further increases the risk of severe toxicity [20].

Diarrhea and enterocolitis (i.e., diarrhea plus pain, bleeding, or inflammation) are the most important complications after ipilimumab (in monotherapy or in combination with PD1 inhibitors), potentially resulting in ICU admission. Furthermore, cases of intestinal perforation have been described and urgent assessment and management are recommended in suspicious cases [21, 22]. The onset of GI symptoms is typically not earlier than 6 weeks after start of treatment [18].

Unspecific symptoms such as fatigue, weakness, nausea, confusion, or headache may lead to the diagnosis of a complex hormonal disorder caused by an endocrine IRAE. The thyroid and pituitary glands are the systems mainly affected in these cases. Symptoms appear, on average, after 9 weeks of treatment [18]. While severe endocrine dysfunctions after PD-1 inhibitors are rare, grade 3 or 4 events were observed in about 5% of ipilimumab-treated patients [16, 17]. Hypotension, arrhythmia, dehydration with oliguria, or electrolyte imbalances may be an indication for ICU admission [23].

The value of autoantibody assays in the management of IRAEs is not well defined. They should, however, be part of diagnostic evaluation and follow-up. In all small retrospective studies with pembrolizumab in advanced melanoma, 40% of patients with thyroid adverse events had detectable autoantibodies against thyroid peroxidase or TSH receptors [24].

Critically ill cancer patients with manifest or suspected IRAE should be managed in strong collaboration between ICU staff, oncologists and organ specialists. A recommendation for incremental workup and treatment is presented in Table 4.

**Table 4:** Work-up of critically ill cancer patients admitted to the ICU suspected for an IRAE

Basic evaluation
(1) When was the treatment with checkpoint inhibitor started and how many doses has the patient already received?
(2) Which is/are the leading symptom/s and when did it/they start?
(3) Which grading definition(s) according to NCI CTCAE is fulfilled?
(4) Rule out important differential diagnosis: pre-existing autoimmune condition, complication of underlying malignancy, infection
(5) What is the patient’s prognosis due to malignancy?
Initial management
(1) ICU monitoring, venous/arterial access, fluid load, vasopressors and oxygen supplementation, ultrasound, and/or CT scan as indicated
(2) Check common laboratory tests: hematology, chemistry (including renal and liver function tests), coagulation, endocrine function, microbial and viral infections, autoantibodies (e.g., ANA, AMA, SMA, LKM1, pANCA, TPOAb, TRAb, TGAb)
(3) If diagnosis of IRAEs is established, initiate steroid therapy at 1–2 mg/kg of body weight OR, if patient is already on steroids, consider increase of dose (up to 5 mg/kg or equivalent)
(4) Involve organ specialists: gastroenterology, endocrinology, and neurology, surgery (if perforation or ileus is suspected)
Advanced support
(1) If symptoms do not improve after 5–7 days, discuss additional immunosuppressive intervention (mycophenolate mofetil, tacrolimus)
(2) Consider endoscopy and colonic biopsies for patients with diarrhea/colitis, or liver biopsy in selected cases
(3) Evaluate specific recommendations for organ dysfunction: -Hormone replacement in endocrine disorders -Infliximab in severe colitis
(4) In responding events slowly taper steroids over 4 weeks; discuss duration of alternative immunosuppression (if needed) with organ specialist
(5) Checkpoint inhibition should be discontinued definitively after grade 3/4 IRAEs

*IRAE* immune-related adverse event, *ANA* antinuclear antibodies, *AMA* antimitochondrial antibodies, *SMA* smooth muscle antibodies, *LKM1* liver kidney microsomal antibodies, *pANCA* perinuclear antineutrophil cytoplasmatic antibodies, *TPOAb* thyroid peroxidase antibodies, *TRAb* TSH receptor antibodies, *TGAb* thyreoglobulin antibodies

## Interstitial pneumonia and pneumonitis

Dyspnea and hypoxemia belong to the leading indications for ICU admissions of cancer patients. Interstitial pneumonia and pneumonitis, both synonymous for lung diseases in which the inflammation process affects the interstitial lung parenchyma, have etiologies including a broad spectrum of infectious and non-infectious causes where the risk for acute lung injury is high [25, 26].

**Fig. 2:**
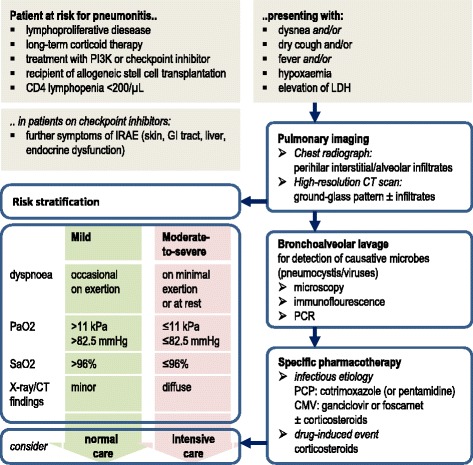
Management of patients suspected or diagnosed with pneumonitis (risk stratification adapted from [26]). *CMV* cytomegalovirus, *LDH* lactate dehydrogenase, *PCP Pneumocystis jirovecii* pneumonia, *PI3K* phosphoinositol-3-kinase

*Pneumocystis jirovecii* and cytomegalovirus are the main infectious agents for pneumonia in immunocompromised patients; for these patients lymphoproliferative malignancies, long-term use of glucocorticoids, lymphocytopenia (CD4 < 200/μL), and allogeneic hematopoietic stem cell transplantation are known risk factors [27]. Guidelines for the management of *P. jirovecii* pneumonia in non-HIV-infected hematological patients were recently updated by the European Conference on Infections in Leukemia (ECIL) [26]. The authors emphasized the need for immediate treatment which should not be delayed by diagnostic procedures. The severity grading which categorizes HIV-positive *P. jirovecii* pneumonia patients into mild, moderate, or severe cases was recommended only for dichotomized use in the non-HIV population (mild versus moderate-to-severe; Fig. 2). This differentiation might be helpful in patient allocation between normal and ICUs as well as in deciding how to administer the antimicrobial agents (orally versus intravenously). High-dose cotrimoxazole (90–120 mg/kg/day, intravenously over ≥14 days) remains the treatment of choice for first-line therapy. An oral route from the beginning is an option only in stable patients with mild disease, which are rarely seen in hematology. Pentamidine (4 mg/kg/d i.v.) or the combination of primaquine (30 mg/d) and clindamycin (3× 600 mg/d) can be considered in patients with contraindications to, or relapsing after, cotrimoxazole. The ECIL authors pointed out that evidence from randomized clinical trials examining the role of adjunctive corticosteroids is available only for HIV-positive patients, but not for the non-HIV population. Therefore, the routine use of corticosteroids in this cohort was not recommended.

The incidence of non-infectious, drug-induced pneumonitis is variable between different agents and studies and has been observed in up to 15% of treated patients (also including the classic cytotoxic agents) [28]. The pathophysiology of lung damage is not fully understood, particularly for some of the newer compounds. Pulmonary toxicity after rituximab has been reported with patterns of organizing, desquamative interstitial, or granulomatous pneumonitis. Dyspnea in patients on TKI for CML may be attributable to pleural or pericardial effusions in rare cases [29, 30]. However, in newly diagnosed patients treated with dasatinib, pleural effusions were observed in 28% of the cases (3% grade ≥3, 12% need for thoracocentesis) [31].

More recently, the therapeutic inhibition of phosphoinositol-3-kinase (PI3K), an enzyme widely expressed in many cell types and involved in intracellular signaling, was observed to be associated with pulmonary toxicity. In a series of clinical trials, patients with chronic lymphocytic leukemia or lymphoma who had received the new PI3K inhibitor idelalisib showed an increased frequency of pneumonitis, some cases of which were fatal [32]. The cumulative incidences in phase 1–3 studies with this compound for cough, dyspnea, and pneumonia were reported to be between 10 and 20%; non-infectious pneumonitis was diagnosed in up to 5% of patients. The mechanism of lung disease in the latter cases remains hypothetical and hypersensitivity reactions, as well as organizing pneumonia, are discussed [33].

Pneumonitis was also reported as a rare immune-mediated complication of checkpoint inhibitor therapy [17, 23, 34]. The highest incidence (5 to 10%) was reported after a combination regimen (ipilimumab plus nivolumab), whereas 2% of these were grade 3 or 4 events [22]. The onset of symptoms was a median of 2.6 months after initiation of therapy in a recently published series of 20 patients who developed pneumonitis after nivolumab [35]. According to the radiographic pattern, the diseases in this cohort were categorized as cryptogenic organizing pneumonia, nonspecific interstitial pneumonia, hypersensitivity pneumonitis, or acute interstitial pneumonia/acute respiratory distress syndrome. Another case report described an organizing pneumonia occurring as a result of transbronchial biopsy in a patient treated with ipilimumab for melanoma [36].

Corticosteroids 1–2 mg/kg/day are the recommended intervention for different types of non-infectious pneumonitis to limit the inflammatory and immune-related reactions. Additional immunosuppression with infliximab or mycophenolate should be considered in patients with IRAE-associated pneumonitis who do not respond to corticosteroids [22].

## Events associated with impaired angiogenesis

Neoangiogenesis is one of the hallmarks of cancer because blood supply is essential for malignant cell proliferation and tumor growth. The development of angiogenesis inhibitors was therefore an obvious aim in cancer research. Bevacizumab, a MAB targeting VEGF was the first antiangiogenetic drug which significantly improved the outcome of patients with metastatic colorectal cancer [37]. Today, bevacizumab is approved for many cancer types, among them frequent malignancies such as breast cancer and (non-small cell) lung cancer. Aflibercept and ramucirumab are more recent angiogenesis inhibitors with proven efficacy in patients with advanced colorectal cancer and gastric cancer, respectively.

Angiogenesis is crucial not only for malignant tissues; the integrity and function of normal cells and tissues depend on blood vessel regeneration as well. This is the background for some important toxicities which have been observed after the administration of antiangiogenetic agents (Table 5). Although the events are mild to moderate in the majority of treated patients, in some cases severe or life-threatening complications have been reported.

**Table 5:** Selected toxicities of antineoplastic pharmacotherapy and antiangiogenetic pathomechanisms (according to [39, 40, 44, 45, 50])

Bevacizumab
→ Arterial hypertension
Decreased endothelial production of nitric oxide with consecutive vasoconstriction Cholesterol embolization syndrome
→ Congestive heart failure
Disruption of physiological coronary angiogenesis Impaired response to pressure overload
→ Arterial thromboembolism
Reduction of anti-inflammatory effects and atherosclerotic instability Impaired proliferation and repair of endothelial cells Endothelial cell dysfunction and exposure of subendothelial collagen Direct platelet activation Inhibition of collateral circulation
Dasatinib
→ Pulmonary hypertension
Reduced hypoxic vasoconstriction Induction of pulmonary endothelial cell apoptosis Induction of reactive oxygen species and consecutive endothelial dysfunction
Dasatinib, nilotinib, ponatinib
→ Cardiovascular events
Metabolic effects: hyperglycaemia, hyperlipidemia Interaction with VEGF receptors Inhibition of KIT and PDGF receptor Inhibition of discoidin domain receptor 1

*VEGF* vascular endothelial growth factor, *KIT* stem cell factor receptor, *PDGF* platelet derived growth factor

Two meta-analyses of randomized trials, each including more than 10,000 patients treated for different cancers with chemotherapy with or without bevacizumab, analyzed the risk for vascular adverse events. The first study revealed an incidence of all-grade arterial thromboembolic complications in bevacizumab-treated patients of 3.3% (relative risk 2.08) with a frequency of severe events (grade ≥3) of 2.0% [38]. Remarkably, the number of thromboembolic events varied greatly between different cancer types. While high-grade events occurred in only 1.0% of breast cancer patients, their incidence was 11.3% in those with lung cancer. The second study showed a significantly increased risk of treatment-related vascular mortality after bevacizumab. The relative risks for fatal pulmonary or GI hemorrhage, GI tract perforation, and cerebrovascular events were 3.96, 3.71, 2.45, and 3.60, respectively [39]. Furthermore, bevacizumab was also found to be associated with hypertension, decreased left ventricular ejection fraction, and congestive heart failure [40, 41].

In addition to bevacizumab, a substantial number of modern TKIs (sunitinib, sorafenib, pazopanib, axitinib, and others) also affect the VEGF pathway and physicians must be aware of potentially associated cardiovascular events in treated patients. Sorafenib and sunitinib, as well as trastuzumab, were shown in a meta-analysis to increase the risk for reduced left ventricular ejection fraction and hypertension; a significantly higher incidence of myocardial infarction was reported with sorafenib treatment [41, 42]. In contrast, no impact of VEGF receptor pathway-relevant TKIs on the frequency of GI tract perforations was found [43].

When imatinib, the first available signaling pathway inhibitor, was introduced in CML, it was regarded as a highly BCR-ABL-specific drug. With both an increase in the number of treated patients and the development of follow-up compounds, a correlation between these agents and their effects on other kinases, including those involved in vascular biology, was identified [44]. Peripheral arterial and cardiovascular diseases were reported from CML patients treated in different TKI trials and from postmarketing populations [44–46], among them events with a toxicity grading of 3 or 4. The potential risk for vascular complications seems to vary between different TKIs, and imatinib may even have protective effects [47]. Due to increasing numbers of thromboembolic adverse events in the follow-up of several ponatinib trials, the European Medicines Agency recommended the drug be avoided in patients who have had a heart attack or stroke in the past and to prescribe it cautiously in general to the benefit–risk profile [48]. Nine cases of moderate-to-severe pulmonary hypertension associated with dasatinib were observed in the French pulmonary hypertension registry [49]. In a preclinical model, dasatinib was found to induce pulmonary endothelial damage which increases susceptibility to pulmonary hypertension [50].

Because thromboembolic complications have a high incidence in cancer patients, the decision of whether an event is caused by the underlying malignancy or by antineoplastic medication may be difficult to make. For antithrombotic treatment, angioplasty, or vascular surgery, however, there are no specific recommendations depending on etiology. Once more, an interdisciplinary approach is required which, for these patients, includes an interventional radiologist and surgeon.

## Conclusions

During the past years, many new compounds have been introduced in clinical cancer treatment, and cellular therapy is a highly dynamic evolving field. However, physicians have also been faced with new side effects associated with these approaches and they must develop strategies to treat affected patients. This process is also reflected by the numerous meta-analyses which have focused on specific adverse events. Treating staff need to be aware of potentially severe complications; critically ill cancer patients after antineoplastic pharmacotherapy implementing new or classic agents should be managed in strong collaboration between ICU staff, oncologists, and organ specialists.

Knowledge about the pathophysiology of new side effects and the recommendations for their diagnostic and therapeutic management are still very limited and must be improved. Strategies to identify patients who are at-risk for different complications are needed. Therefore, additional clinical and preclinical research in this field is urgently needed.

## Abbreviations

BAB: Bispecific antibody
CAR: Chimeric antigen receptor
CML: Chronic myeloid leukemia
CNS: Central nervous system
CRS: Cytokine release syndrome
CTCAE: Common Terminology Criteria for Adverse Events
ECIL: European Conference on Infections in Leukemia
EGFR: Epidermal growth factor receptor
GI: Gastrointestinal
HIV: Human immunodeficiency virus
ICU: Intensive care unit
IL: Interleukin
IRAE: Immune-related adverse event
MAB: Monoclonal antibody
NCI: National Cancer Institute
PI3K: phosphoinositol-3-kinase
PD-1/2: programmed cell death receptor 1/2
TKI: Tyrosine kinase inhibitor
VEGF: Vascular endothelial growth factor
